# Factors Related to the Attitude of North Korean Refugees Towards People with Mental Illness

**Published:** 2018-07

**Authors:** Jin-Won NOH, Jong-Min WOO, Hyunchun PARK, Sun Jae JUNG, Yejin LEE, Young Dae KWON

**Affiliations:** 1. Dept. of Healthcare Management, College of Health Science, Eulji University, Seongnam, Korea; 2. Global Health Unit, Dept. of Health Sciences, University Medical Centre Groningen, University of Groningen, Groningen, The Netherlands; 3. Korea Employee Assistance Professionals Association, Seoul, Korea; 4. Gyeonggi Public Health Policy Institute, Seongnam, Korea; 5. Dept. of Epidemiology, Harvard T.H. Chan School of Public Health, Boston, USA; 6. Dept. of Humanities and Social Medicine, College of Medicine and Catholic Institute for Healthcare Management, The Catholic University of Korea, Seoul, Korea

**Keywords:** Mental illness, Attitude, Mental health knowledge, North Korean

## Abstract

**Background:**

The purpose of this study was to explore the level of mental health knowledge among North Korean refugees residing in South Korea and to analyze the factors related to their attitude towards people with mental disorders.

**Methods:**

The survey was conducted on 150 people and the analysis included results from 138 participants in 2013. A multiple linear regression analysis was conducted to analyze the factors related to the attitude towards people with mental illness.

**Results:**

The education level attained in South Korea and the duration of stay in the South were effective factors associated with anti-authoritarianism. Age, marital status, education level in the North, and level of mental health knowledge were significant factors for benevolence. Time spent in South Korea and knowledge of mental health played a significant role in determining their attitude towards social restrictiveness (*P*=0.014).

**Conclusion:**

The knowledge of mental illness in refugees was associated with their attitude towards people with mental illness. This study suggests the need to educate refugees on mental illness to enhance their attitudes.

## Introduction

In the last three decades, war, famine, and political struggles have caused an increase in forced migration worldwide. In 1970, there were approximately 2.5 million refugees. This number increased to 21.3 million in 2015 (1). The path of refugees to reach their new settlement is filled with risks and stressors. In the immigration process, most refugees experience varying degrees of physical and psychological trauma, which can have a significant effect on health problems (2). These traumatic events have a number of causes, one of which includes previous traumatic exposure in their homelands (3). The study of overcoming old traumatic events and adapting to new settlements has become an important social issue in many countries (4–6).

There are various factors that contribute to refugees’ adaptation to the host countries, including socio-economic environment and individual capabilities, and mental health which is also considered an important factor (7, 8). In particular, North Korean refugees suffer from physical and psychological trauma caused by life-threatening environmental factors during the process of fleeing the North and entering South Korea through a third country (9, 10), and many experience post-traumatic stress disorder (11). North Korean refugees, like other typical refugees, also undergo extreme stress due to economic hardships, cultural differences, separation from family, language barriers, and discrimination while settling in a new society (12–14). In many cases, these refugees suffer from mental health problems such as anxiety or depression (15–20).

Mental health issues among resettling refugees are likely to be caused by negative experiences prior to or during migration. In addition, psychosocial factors experienced by the immigrants at the place of resettlement also play an important role, such as a lack of sense of belonging, financial difficulties, discrimination, and insufficient information on mental health (13). Many social and economic factors that affect mental health are not easy to overcome by individual efforts alone. However, the problems due to ignorance of mental health can be partly improved by institutional support and individual acquisition of knowledge. In social psychology, knowledge and attitude are considered useful factors for understanding and changing human behavior (16). The more health knowledge an individual has, the higher the level of self-efficacy (17). Psychiatric consultation and treatment are essential to overcome mental health problems among North Korean refugees, who have extremely limited access to psychiatric care. North Korean refugees are especially reluctant to even visit psychiatrists (18). Lack of knowledge regarding mental health and negative attitudes toward the mentally ill are the main causes of limited access to psychiatric treatment (19).

This study focused on the level of knowledge about mental health among North Korean refugees who reside in South Korea and analyzed the factors underlying their attitudes toward people with mental disorders. Further, the findings may be applicable to the mental health of other refugees that migrated to different nations.

## Methods

### Data and Subjects

North Korean refugees are reluctant to participate in self-rating surveys via questionnaires due to sensitivity towards revealing their identity. Therefore, the survey participants were selected using the snowball sampling method assisted by professional consultants working at the Seoul and Gyeonggi Province regional branches of Hana Center, an organization that provides support to North Korean refugees in the South. The survey was conducted with those who agreed to participate in the study. Random sampling was difficult, but the sample was selected according to the sex ratio (70.7% females, 29.3% males) and age structure (ages 30 to 50 account for 70% of the population) of the North Korean refugee population based on the 2013 Ministry of Unification data on North Korean refugees. The main survey was conducted from August 1st to 30th, 2013, including 150 participants. The analysis included results from 137 participants, excluding 13 with incomplete response to questionnaires.

This study was reviewed and approved in advance by the Institutional Review Board of Inje University (IIT-2013-287). The study was conducted under informed consent of the study subjects and following an approved protocol.

### Variables and Measurements

#### Attitude towards people with mental illness

This study used the Community Attitudes toward the Mentally Ill (CAMI) tool developed by Taylor and Dear (19) and translated into Korean (20) CAMI consisted of 40 questions, including 10 questions for each of the following four sub-categories: anti-authoritarianism, benevolence, social restrictiveness, and community mental health ideology. All questions were scored on a five-point Likert scale. This tool has been verified for validity and reliability (19, 20). The scores of Cronbach’s α, the index of reliability, were 0.68 for anti-authoritarianism, 0.76 for benevolence, 0.80 for social restrictiveness, and 0.88 for community mental health ideology.

### Mental health knowledge

This study assessed the level of mental health knowledge by measuring the knowledge of schizophrenia. In previous studies, the usual components of knowledge were defined as causes, symptoms, drugs, treatments, and recurrence (21, 22). We adopted a tool developed by Lim and Ahn (23) based on the aforementioned five key components and has been used in many previous studies. This tool was translated and independently back translated already (23). The reliability of this tool at the time of its development was Cronbach’s alpha = 0.81.

### Socio-demographic characteristics and defection-related variables

Demographic variables included age, sex, and marital status. Socio-economic variables were: the last level of education in North Korea, the level of education obtained in South Korea, religion, and monthly income. Finally, defection-related variables included the time lapsed since leaving the North and entering the South.

### Statistical Analysis

We calculated descriptive statistics using categorical percentages, means, and standard deviations in relation to the subject’s demographic and socio-economic characteristics; mean and standard deviation were used in relation to mental health knowledge and attitude towards the mentally ill. A multiple linear regression analysis was conducted in order to analyze factors related to the attitude towards people with mental illness. Stata 13.1 (Stata Corp LP, College Station, Texas) was used to analyze data, and the significance was defined as *P*<0.05 (two-tailed).

## Results

The average age of the study participants was 44.9 yr. A total of 60.6% of the subjects were females, and 49.6% were married. More than half (59.9%) had graduated secondary school in North Korea, and 58.7% did not receive additional education in the South. Approximately two-thirds (67.2%) were religious, and 87.4% had a monthly income less than 2 million Korean won. On average, 9.4 yr had passed since the participants left North Korea, and an average of 6.8 yr lapsed since they entered the South. On average, the participants scored 12.8 points out of 28 on the mental health knowledge questionnaire. The average scores regarding their attitude towards people with mental illness were 30.6 points out of 50 for anti-authoritarianism, 32.4 out of 50 for benevolence, 29.0 out of 50 for social restrictiveness, and 32.2 out of 50 for community mental health ideology. Thus, the participants scored the highest points for benevolence (Table 1). Examination of the factors underlying their attitude towards mentally ill patients showed that education obtained in South Korea and the length of time spent in the South were significant factors contributing to anti-authoritarianism (*P*=0.008). Participants who obtained an educational level of university graduate or higher in the South (compared to those who did not get additional education) or participants who had lived in the South for a longer period of time showed a more positive attitude towards anti-authoritarianism. Age, marital status, level of education in the North, and level of mental health knowledge were significant related factors related to benevolence. Older, single (compared to divorced and widowed), with secondary school or university education and above (compared to those with an education level of primary school or lower), and/or higher scores on the mental health knowledge test, suggested a more positive attitude towards benevolence. Time spent in South Korea and the level of mental health knowledge, were significant (*P*=0.014) factors shaping social restrictiveness.

**Table 1:** Characteristics of the study population

***Variable***	***Category***	***N (%)*** ***mean ± SD (min, max)***
Age (yr)		44.9 ± 15.5 (19, 87)
Sex	Male	54 (39.4%)
	Female	83 (60.6%)
Marital status	Single	37 (28.7%)
	Married	64 (49.6%)
	Other (divorced/widowed/separated)	28 (21.7%)
Education in North Korea	People’s school	9 (6.6%)
	Senior middle school	82 (59.9%)
	College	26 (19.0%)
	University or higher	20 (14.6%)
Education in South Korea	None	81 (59.6%)
	Elementary/middle school	6 (4.4%)
	High school	15 (11.3%)
	College or higher	34 (25.0%)
Religion	Religious	92 (67.2%)
	Not religious	45 (32.2%)
Monthly income (Korean won)	Less than 1 million (< 890 USD)	52 (43.7%)
	1 million – 2 million (890 – 1,780 USD)	52 (43.7%)
	More than 2 million (≥ 1,780 USD)	15 (12.6%)
Duration since entry into South Korea (years)		6.8 ± 3.4 (0, 15)
Duration since escape from North Korea (years)		9.4 ± 4.8 (0, 25)
Knowledge-related correct answer rate (0 – 28)		12.9 ± 4.4 (3, 24)
Anti-authoritarianism (10 – 50)		30.6 ± 3.3 (23, 42)
Benevolence (10 – 50)		32.4 ± 3.0 (25, 41)
Social restrictiveness (10 – 50)		29.0 ± 3.1 (18, 35)
Community mental health ideology (10 – 50)		32.2 ± 3.8 (22, 43)

Abbreviations: SD, standard deviation; USD, United States dollar

Participants who had been in South Korea longer or who had a higher level of mental health knowledge showed a more positive attitude towards social restrictiveness. There were no significant factors contributing to community mental health ideology (Table 2).

**Table 2:** Analysis off actors shaping attitudes toward people with mental illness

***Variable***	***Category***	***Anti-authoritarianism***			***Benevolence***			***Social restrictiveness***			***Community mental health ideology***		
		**Coef.**	**95%**	**CI**	**Coef.**	**95%**	**CI**	**Coef.**	**95%**	**CI**	**Coef.**	**95%**	**CI**
Age (yr)		0.03	−0.03	0.09	0.07^*^	0.01	0.12	0.02	−0.03	0.08	0.05	−0.02	0.13
Sex	male (ref)												
	female	−0.30	−1.79	1.19	−0.38	−1.71	0.95	−0.56	−1.93	0.82	−0.71	−2.52	1.09
Marital status	single (ref)												
	married	0.66	−1.11	2.43	0.24	−1.35	1.82	0.61	−1.03	2.25	1.06	−1.08	3.20
	other (divorced/widowed/separated)	−0.72	−2.85	1.41	−2.10^*^	−4.01	−0.18	−1.36	−3.31	0.60	−1.42	−4.01	1.16
Education in North Korea	people’s school (ref)												
	senior middle school	−2.20	−6.02	1.63	−0.17	−3.55	3.20	−0.57	−4.11	2.96	−3.98	−8.64	0.69
	college	0.35	−1.41	2.12	2.43^†^	0.84	4.01	0.39	−1.26	2.05	1.39	−0.83	3.62
	university or higher	0.15	−2.19	2.48	3.39^†^	1.31	5.47	−0.21	−2.36	1.93	1.82	−1.06	4.70
Education in South Korea	none (ref)												
	elementary/middle school	1.00	−3.72	5.72	0.67	−3.48	4.83	−3.88	−8.22	0.47	1.53	−4.16	7.23
	high school	0.59	−1.74	2.91	0.07	−2.02	2.17	1.14	−0.99	3.27	−0.22	−3.02	2.58
	college or higher	2.56^†^	0.71	4.42	0.09	−1.50	1.68	1.23	−0.43	2.89	0.48	−1.76	2.72
Religion	religious (ref)												
	non-religious	1.28	−0.24	2.80	0.25	−1.13	1.64	0.56	−0.84	1.96	0.87	−0.98	2.72
Monthly income (Korean won)	less than 1 million (< 890 USD) (ref)												
	1 million – 2 million (890 - 1,780 USD)	0.55	−0.92	2.03	0.88	−0.46	2.23	0.67	−0.69	2.03	0.71	−1.06	2.48
	more than 2 million (≥ 1,780 USD)	1.05	−1.11	3.21	1.78	−0.13	3.70	−0.47	−2.46	1.52	0.90	−1.76	3.56
Duration after escape from North Korea		−0.20	−0.45	0.04	−0.07	−0.29	0.14	−0.13	−0.35	0.09	−0.18	−0.48	0.11
Duration after entry into South Korea		0.45^†^	0.12	0.78	0.01	−0.28	0.31	0.41^*^	0.11	0.71	0.34	−0.06	0.73
Knowledge of mental health		0.11	−0.05	0.27	0.17^*^	0.02	0.32	0.19^*^	0.04	0.34	0.13	−0.06	0.33

* *P*<0.05,

† *P*<0.01

Abbreviations: Coef., coefficient; CI, confidence interval; ref, reference; USD, United States dollar

## Discussion

The aim of this study was to explore the level of mental health knowledge among North Korean refugees residing in South Korea and analyze the related factors shaping their attitude towards people diagnosed with mental disorders. A significant association was found between the knowledge and specific domains of attitude such as benevolence and social restrictiveness towards people with mental illness. The knowledge-related correct answer score showed a mean value of 12.82 (SD 4.40), which is relatively low compared with the results of other studies conducted among South Koreans (23, 24). It is hard to obtain information related to mental illness or mentally ill patients among people living in North Korea, due to the strict governmental regulation of media. Media in North Korea are supposed to report only positive aspects of the society, and not the negative realities including the presence of mentally ill patients. In North Korea, people with mental illness are mostly admitted to ‘hospital 49,’ which is hard to access by the general population. Therefore, most of the people in North Korea are not well informed about mental illness, including schizophrenia (25). However, North Korean refugees showed an increased level of mental health knowledge following several years of education in South Korea (26). In this study, the attitude-related correct answer score was 30.6 for anti-authoritarianism, 32.4 for benevolence, 29.0 for social restrictiveness, and 32.2 for community mental health ideology. Overall scores were different from those of South Koreans: 33.5 for anti-authoritarianism, 22.8 for benevolence, 31.1 for social restrictiveness, and 26.9 for community mental health ideology (27).

North Korean refugees had relatively negative attitudes toward anti-authoritarianism. North Korea has a Soviet-style authoritarian political system, in which people are accustomed to classify other people with potential rank (28). Thus, North Korean refugees may have increasingly negative preconceptions and an authoritarian attitude towards people living with mental illness. However, the results showed an increased positive attitude towards anti-authoritarianism, as refugees attain higher education and after a longer residency period in South Korea. Thus, it appears that the knowledge acquired by the refugees from South Korean society influenced their attitude towards mentally ill patients. North Korean refugees also had increasingly positive thoughts about benevolence than South Korean residents, and scored higher on community mental health ideology. North Korean refugees are offered support from the government or private organizations during the migration from the North to the South Korea for resettlement, suggesting that the experience contributes to benevolence towards the mentally ill, or increases empathy for people with mental illness.

Participants who had a higher level of education in the South and had lived longer in the South showed a positive attitude towards anti-authoritarianism. Besides, participants with a longer period of residence in the South showed a positive attitude towards unrestricted social activities. The findings suggest that the educational attainments of North Korean refugees in the South, and the information gained in everyday life altered their attitude towards mentally ill patients in a positive direction. North Korean refugees under psychiatric treatment showed a more positive attitude towards people with mental illness (29). Therefore, the promotion and education of mental health via psychiatric treatment and knowledge can help improve the attitudes of North Korean refugees towards mentally ill patients. It is necessary to increase the mental health educational opportunities immediately after entry into a new environment and at the initial stages of settlement to improve access to mental health services. Mental health services at the initial stages of settlement have encouraged the refugees to contact the providers and reduce the difficulty in finding services (30). Similar findings were reported in previous studies involving African refugees (31).

This study had certain limitations. As the participants were recruited using snowball sampling, they may not accurately represent all refugees from North Korea. Thus, it is important to be cautious when generalizing the results to the entire North Korean refugee population. Second, as the study was conducted only among North Korean refugees without South Korean controls, it was hard to compare the results directly. In other words, comparing our results to previous studies with South Korean residents may be influenced by differences in basic characteristics. Further, it is unclear whether an increase in knowledge directly alters the attitudes because this study did not examine causal relationship. A further study should be conducted with study participants representing the actual population in North Korean refugees and should include comparable controls in the South Korean population.

## Conclusion

The knowledge of mental illness in refugees shaped their attitude towards people with mental illness. A longer period of residence was correlated with a more positive attitude towards the mentally ill. This study underscores the need to educate refugees on mental illness to enhance their attitudes and facilitate mental health management of citizens. Specifically, it is necessary to increase the educational opportunities for mental health at the initial stages of settlement to improve access to mental health services.

## Ethical considerations

Ethical issues (Including plagiarism, informed consent, misconduct, data fabrication and/or falsification, double publication and/or submission, redundancy, etc.) have been completely observed by the authors.
